# Performance of Electropun Polyacrylonitrile Nanofibrous Phases, Shown for the Separation of Water-Soluble Food Dyes via UTLC-Vis-ESI-MS

**DOI:** 10.3390/nano7080218

**Published:** 2017-08-10

**Authors:** Pimolpun Niamlang, Pitt Supaphol, Gertrud E. Morlock

**Affiliations:** 1 Department of Materials Engineering, Faculty of Engineering, Rajamangala University of Technology Rattanakosin, 96 Mu 3 Phutthamonthon Sai 5 Road, Salaya, Phutthamonthon, Nakorn Pathom 73170, Thailand; pimolpun.kam@rmutr.ac.th; 2 Institute of Nutritional Science, Chair of Food Science, Justus Liebig University Giessen, Heinrich-Buff-Ring 26-32, 35392 Giessen, Germany; 3 The Petroleum and Petrochemical College, Chulalongkorn University, Bangkok 10330, Thailand; pitt.s@chula.ac.th

**Keywords:** nanomaterials, mass spectrometry, planar chromatography, ultrathin-layer chromatography, quantification, electrospinning process

## Abstract

Research in the miniaturization of planar chromatography led to various approaches in manufacturing ultrathin-layer chromatography (UTLC) layers of reduced thickness (<50 µm) along with smaller instrumentation, as targeted in Office Chromatography. This novel concept merges 3D print & media technologies with miniaturized planar chromatography to realize an all-in-one instrument, in which all steps of UTLC are automated and integrated in the same tiny device. In this context, the development of electrospun polyacrylonitrile (PAN) nanofiber phases was investigated as well as its performance. A nanofibrous stationary phase with fiber diameters of 150–225 nm and a thickness of ca. 25 µm was manufactured. Mixtures of water-soluble food dyes were printed on it using a modified office printer, and successfully separated to illustrate the capabilities of such UTLC media. The separation took 8 min for 30 mm and was faster (up to a factor of 2) than on particulate layers. The mean *hR*_F_ values ranging from 25 to 90 for the five food dyes were well spread over the migration distance, with an overall reproducibility of 7% (mean %*RSD* over 5 different plates for 5 dyes). The individual mean plate numbers over 5 plates ranged between 8286 and 22,885 (mean of 11,722 over all 5 dyes). The single mean resolutions *R*_S_ were between 1.7 and 6.5 (for the 5 food dyes over 5 plates), with highly satisfying reproducibilities (0.3 as mean deviation of *R*_S_). Using videodensitometry, different amounts separated in parallel led to reliable linear calibrations for each dye (*sdv* of 3.1–9.1% for peak heights and 2.4–9.3% for peak areas). Coupling to mass spectrometry via an elution head-based interface was successfully demonstrated for such ultrathin layers, showing several advantages such as a reduced cleaning process and a minimum zone distance. All these results underline the potential of electrospun nanofibrous phases to succeed as affordable stationary phase for quantitative UTLC.

## 1. Introduction

Thin-layer chromatography (TLC) was developed in the 1950s and widely used for a variety of applications including the identification of drugs and toxic substances in biological fluids, the monitoring of water supplies for pesticides, the analysis of pharmaceutical products, and the evaluation of the flavor potential of plant materials [1]. Normal phase separations on silica gel 60 adsorbent are best established and most widely used in TLC [2,3]. A substantial change came in the 1970s with the introduction of high-performance TLC (HPTLC) plates. Compared to TLC plates, HPTLC plates have thinner layers containing a smaller size of particles with a more homogeneous distribution, providing shorter migrations distances, faster separations, and lower reagent and mobile phase consumptions [2,3].

Ultrathin-layer chromatographic (UTLC) plates were introduced in 2001 to improve the efficiency of given plates and adsorbents in terms of sensitivity, analysis times, and the amount of consumables [4,5]. First, UTLC plates were made by coating a glass substrate with a monolithic silica gel, creating a 10-µm thick adsorbent layer characterized by 1–2 µm macropores and 3–4 nm mesopores. Separations on UTLC plates were reported to be faster and required smaller reagent and sample volumes than on TLC and HPTLC plates. In most cases, UTLC plates had a lower limit of detection. In contrast to all these benefits, the separation number and resolution between zones were often worse due to a shorter migration distance and lower available specific surface area [5]. UTLC plates have been shown to provide a better interface in coupled TLC-mass spectrometry (MS), where the thinner adsorbent layer on UTLC plates improved the sensitivity of TLC-atmospheric pressure matrix-assisted laser desorption ionization (AP MALDI)-MS by 10–100 times over HPTLC plates [6]. Binder materials may have an impact on such sensitive devices and on the efficiency of separations due to the introduction of heterogeneous interaction sites. Hence, it is advantageous that electrospinning of the stationary phases in UTLC allows the production of binder-free UTLC plates and gives the scientist control of stationary phase mat thicknesses and chemical functionality present using a minimal amount of materials (about 1 mL of polymer solution) [7,8,9]. 

Though electrospinning of an polyacrylonitrile (PAN) nanofibrous stationary phase has already been shown [7], applications and MS hyphenations are rare. In this study, the manufactured electrospun PAN nanofiber mat was used and evaluated as stationary phase for UTLC. The performance of the application of a water-soluble food dye mixture by a modified office printer was investigated. Per se dyes are ideal analytes for evaluation of manufactured plates and for their optimization. Following method development and successful separation, the mass spectra of the dyes on such nanofibrous stationary phases were recorded. The zones were directly eluted into the mass spectrometer using a TLC-MS interface.

## 2. Experimental

### 2.1. Materials

Platinum used for scanning electron microscopy (SEM) was obtained from SPI-Chem (West Chester, PA, USA). Aluminum foil (with a thickness of 50 µm) was delivered by Korff, Oberbipp, Switzerland. PAN (ca. 55,500 Da average molecular weight, containing 8.6% methyl acrylate comonomer, *w*/*w*) was obtained from Thai Acrylic Fibre, Lumpini Bangkok, Thailand. Dimethylformamide (purity ≥ 99.8%) and methanol (purity ≥ 99.8%) were purchased from Sigma Aldrich, Buchs, Switzerland. Toluene (purity ≥ 99.8%) and HPTLC silica gel 60 aluminum foils and HPTLC plates silica gel 60 CN (cyano phase) were purchased from Merck, Darmstadt, Germany. Ammonium hydroxide (NH_3_ 25%, analytical grade) was obtained from VWR, Darmstadt, Germany. The following water-soluble food dyes were used: tartrazine (E 102; Carl Roth, Karlsruhe, Germany), chrysoine resorcinol (E 103; Sigma Aldrich) quinoline yellow (E 104; Ringe and Kuhlmann, Hamburg, Germany), fast yellow AB (E 105, purity ≥ 95%; Sigma Aldrich), orange GGN (E 111; Schuhmann and Son, Karlsruhe, Germany), erythrosine (E 127, purity > 85%; Ringe and Kuhlmann) and brilliant blue FCF (E 133, purity > 80%; Schuhmann and Son).

### 2.2. Fabrication of Electrospun PAN Nanofiber Phases

The spinning solution of 12 wt % was prepared by dissolving the PAN in dimethylformamide under mechanical stirring for about 1 h at room temperature (25 ± 1 °C). The solution was then loaded into a standard 10 mL glass syringe. A 20-gauge, blunt-ended stainless steel needle (an outer diameter of 0.91 mm) was used as nozzle. Both the syringe and the needle were tilted about 45° from a horizontal baseline. A sheet of aluminum foil was wrapped around a rotating cylinder (an outer diameter of ca. 15 cm and a rotational speed of ca. 1000 rpm) and placed at a fixed distance of 10 cm from the needle tip. The needle was connected to the emitting electrode of positive polarity of an ES30P-5W power supply (Gamma High Voltage Research, Ormond Beach, FL, USA). The electrical potential was fixed at 16 kV. The electrospun PAN nanofiber mat was collected continuously for 90 min. Prior to further use, this fiber mat was placed in vacuo at room temperature (25 ± 1 °C) at least 24 h to remove the solvent [10,11].

The fiber mat was cut from the middle part of the aluminum foil into the size of 3 cm × 4 cm to ensure a uniform thickness of the cut parts used as stationary phase. The morphology of the electrospun PAN nanofiber phase was inspected by a JSM-6400 scanning electron microscope (JEOL, Tokyo, Japan). Each specimen was coated with a thin layer of platinum prior to the SEM observation. The diameters of the individual fiber segments within each specimen were measured directly from the SEM images using ImageJ software (National Institutes of Health, Bethesda, MD, USA). No fewer than 50 diameters were determined on different fiber segments and the average value was calculated.

### 2.3. Standard Solutions

For a standard mixture, five water-soluble food dyes (25 mg each of E 127 and E 133; 50 mg each of E 104, E 111 and E 102) were dissolved in a 10 mL measuring flask with methanol and filled up to the mark with methanol (2.5 µg/µL). For MS, individual methanolic dye solutions were used (2.5 or 5.0 µg/µL).

### 2.4. Application

The standard mixture solution was printed using a modified (almost completely demounted) Canon printer (Pixma iP 3000 Bubble Jet printer; Canon, Krefeld, Germany) [12]. The electrospun PAN nanofiber phase of 3 cm × 4 cm was loaded onto the CD tray of the printer. The bands were printed as 3 mm bands, normally in band widths between 0.1 and 0.3 mm. The print patterns were drawn in vector illustration software (CorelDraw, Corel, Unterschleißheim, Germany) with a distance from the lower edge of 5 mm, a distance from the left side of 5 mm, and an absolute distance between bands of 1.5 mm. For calibration, 3–9 nL/band of the standard mixture solution were applied on the plate (band heights were 0.2 mm to 0.6 mm).

### 2.5. Chromatography

Development was performed in a homemade micro-chamber (4.5 cm × 1.5 cm × 5.5 cm, height × width × length) made of glass with a mixture of methanol, toluene and ammonium hydroxide 25% (40:57:3, *v*/*v*/*v*). The migration distance was 30 mm from the lower plate edge and the migration time was 8 min. Thereafter, the electrospun PAN nanofiber phase was dried in a flow of air for 10 s.

### 2.6. Documentation and Videoevaluation

For documentation of the tiny electrospun PAN nanofiber phase, the images were captured using a high-resolution flatbed scanner (CanoScan 9000F, Canon, Krefeld, Germany). The VideoScan software (CAMAG, Muttenz, Switzerland) was used for linear calibration via peak area and peak height. The respective image taken was processed using a Savitsky Golay filter (order 2 and width of mostly 17), the lowest slope as the baseline correction mode, and different filters for evaluation. The Sorbfil TLC Videodensitometer (Jsc Sorbpolymer, Nicosia, Cyprus) was also used to evaluate spot areas on a TLC plate image with the construction of a densitogram based on the difference between the track intensity and the background intensity.

### 2.7. Characterization by Electrospray Ionization Mass Spectrometry (ESI-MS)

For the recording of ESI-MS, the electrospun PAN nanofiber phase was eluted online using the TLC-MS Interface (CAMAG) equipped with the 4 mm circular elution head. This elution head-based interface was coupled to the electrospray ionization (ESI) interface of the single-quadrupole mass spectrometer (G1956B MSD, Agilent, Waldbronn, Germany). In the transfer tube from the interface to the ESI source, an inline filter (A-356 containing a 0.5 µm PEEK-Frit Blue UP A-703; both Upchurch Scientific, Techlab, Erkerode, Germany) was integrated. The selected zone on the fiber mat was eluted with methanol at a flow rate of 50 µL/min. The MS system was operated in full scan mode between *m/z* 110 and *m*/*z* 800 in the negative or positive ionization mode with the following parameters: capillary voltage: −/+ 4 kV; nebulizer gas pressure: 40 psig; drying gas temperature: 300 °C; drying gas flow rate: 10 L/min; fragmentation voltage: 100 V; gain: 1; threshold: 10; step size: 0.2. Data processing for MS measurements was carried out with LC/MSD ChemStation software Rev. B. 02.01-SR2 (Agilent, Santa Clara, CA, USA).

## 3. Results and Discussion

### 3.1. Characterization of Electrospun PAN Nanofibers

**Figure 1:**
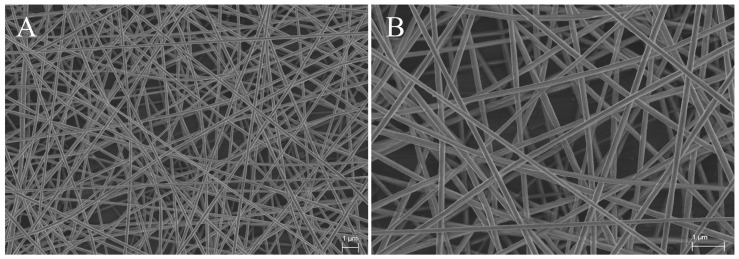
Selected SEM images of a typical electrospun PAN nanofiber phase taken at a magnification of 5000× (**A**) and 10,000× (**B**).

The electrospun PAN nanofiber phases were fabricated using a 12 wt % PAN solution in dimethylformamide according to the procedure reported previously [11]. The mean diameter (*n* = 50) of the individual fibers was 224 ± 65 nm, and the mean thickness (*n* = 50) was 26 ± 3 µm. The morphology of the nanofiber phase was uniform (Figure 1). The cross-sections of the fibers were round, and their surfaces smooth. The resulting nanofiber phases (without binder) were mechanically stable (like tights) and suited for the UTLC workflow.

### 3.2. Comparison of Migration Velocities on Three Different Phases

**Figure 2:**
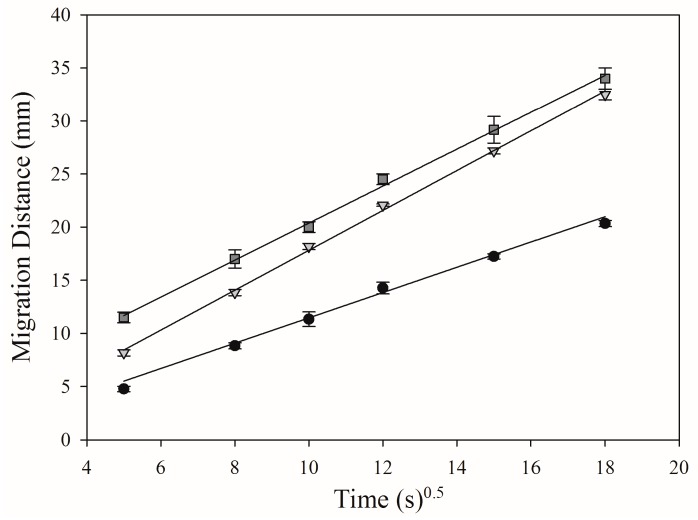
Comparison of mean mobile phase velocities with standard deviation (SD, *n* = 3) on HPTLC foil silica gel 60 (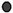), HPTLC plate silica gel CN (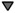) and electrospun PAN nanofiber phase (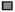).

The mean migration velocity of the mobile phase on electrospun PAN nanofibers was compared with that on two commercially available HPTLC silica gel 60 and cyano phases. The Lucas–Washburn Equation (1) [7,13,14] was used to describe the liquid transportation through porous media as follows:

*Z_f_*^2^ = (*γ*·*R*·*T*·*cosθ*)/2*η* (1)

where *Z_f_* is the migration distance of the solvent front, *T* the migration time, *γ* the surface tension of the mobile phase, *θ* the contact angle of the mobile phase with the stationary phase, *R* the effective pore radius, and *η* the viscosity of the mobile phase [7]. Heptane was selected as the mobile phase in this study due to its contact angle of 0° with the electrospun PAN nanofiber phase and HPTLC cyano phase. The obtained linear regressions fit well with the Lucas–Washburn equation (*r*^2^ > 0.99), showing that the electrospun PAN nanofiber phase had the fastest mobile phase migration (Figure 2) and was faster by a factor of up to 2, if compared to particulate adsorbents.

### 3.3. Efficiency and Performance of the Electrospun PAN Nanofiber Phase

**Figure 3:**
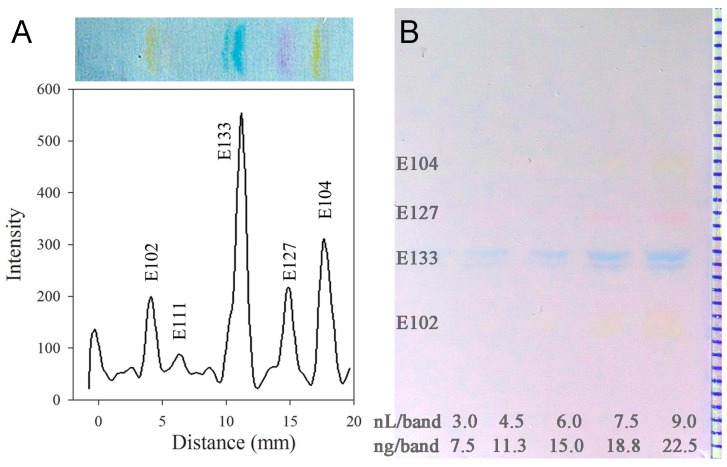
Videodensitogram and chromatogram (enhanced) showing the separation of a water-soluble food dye mixture (**A**) and a typical chromatogram used for calibration (**B**) on the electrospun PAN nanofiber phase (right: millimeter scale).

The dye volumes applied as 3 mm bands on the electrospun PAN nanofiber phase using a modified Canon printer [12] were estimated to range between 3 and 9 nL/band (dependent on the selected band heights between 0.2 and 0.6 mm), which was equivalent to amounts of 8 to 23 ng/band [15,16]. For their separation, eight different solvent selectivities were investigated and adjusted in the elution strength. Toluene showed the best selectivity and was mixed with methanol to adjust the elution strength. The ratios 1:9 to 1:1 were investigated with additions of acetic acid or ammonium hydroxide 25%. The development with a mixture of methanol, toluene and ammonium hydroxide 25% (40:57:3, *v*/*v*/*v*) was found to separate sufficiently the 5 water-soluble food dyes (Figure 3). The migration time was 8 min for a 30 mm migration distance that was limited due to the size of the homemade microchamber with a height of 4.5 cm.

For 5 separations on the electrospun PAN nanofiber phase, the mean *hR*_F_ value (*R*_F_ × 100), the mean plate number *N* according to Equation (2), and the mean resolution *R*_S_ between adjacent zones according to Equation (3) were calculated for each water-soluble food dye. In Equation (2), *Z_S_* is the migration distance of a peak and *w* is its peak width on the basis. In Equation (3), *Z_S_*_1_ and *Z_S_*_2_ are the migration distances, and *w*_1_ and *w*_2_ the peak widths on the basis of the two adjacent peaks 1 and 2, respectively.

*N* = 16 (*Z_S_*/*w*)^2^ (2)

*R*_S_ = 2 (*Z_S_*_2_ − *Z_S_*_1_)/(*w*_2_ + *w*_1_) (3)

The mean *hR*_F_ values of the food dyes ranged from 25 to 90 and were well spread over the migration distance (Table 1, Figure 3). Their overall mean precision was 7% (%*RSD*, *n* = 5, over all 5 food dyes on 5 different plates), showing individual mean precisions between 1 and 16%. The overall mean plate number *N* (over all 5 food dyes for 5 plates) was 11,722, with individual mean plate numbers *N* between 8286 and 22,885. The overall mean resolution *R*_S_ was 4.5 (over all 5 food dyes for 5 plates), whereby the individual mean resolutions *R*_S_ were between 1.7 and 6.5. Their reproducibilities were highly satisfying (0.3 as mean deviation of *R*_S_). For videodensitometry, the visible image was loaded into the VideoScan software, the Sorbfil TLC Videodensitometer (Jsc Sorbpolymer), and the assigned tracks were transformed into densitograms. For conversion, the grey scale pixels within the lines of a track were summed, which generated one data point. The baseline was automatically set using the lowest slope mode. Electronic filters were used to isolate different colors. The red filter was chosen for evaluation of the blue dye (E 133) as well as the blue filter for evaluation of the red (E 127) and yellow dyes (E 102, E 104 and E 111). The digital evaluation of the yellow dyes was more challenging compared to the blue or red dyes, as those yellow dyes were more difficult to see on the white layer background (less contrast, Figure 3B). On the manufactured electrospun PAN nanofiber phase, the linear calibrations of water-soluble food dyes obtained by videoevaluation resulted in precisions of the linear calibration functions of 3.1–9.1% (mean %*RSD* 5.7%) for peak heights and 2.4–9.3% (mean %*RSD* 5.5%) for peak areas (Table 1). Hence, the quality (characterization by SEM), good efficiency (separation time and resolution), and performance (precisions and linear calibrations) of the electrospun PAN nanofiber phase for quantitative studies on such upcoming layer materials was successfully demonstrated, if compared to a powerful HPTLC method [17].

### 3.4. Identification by Mass Spectrometry

**Figure 4:**
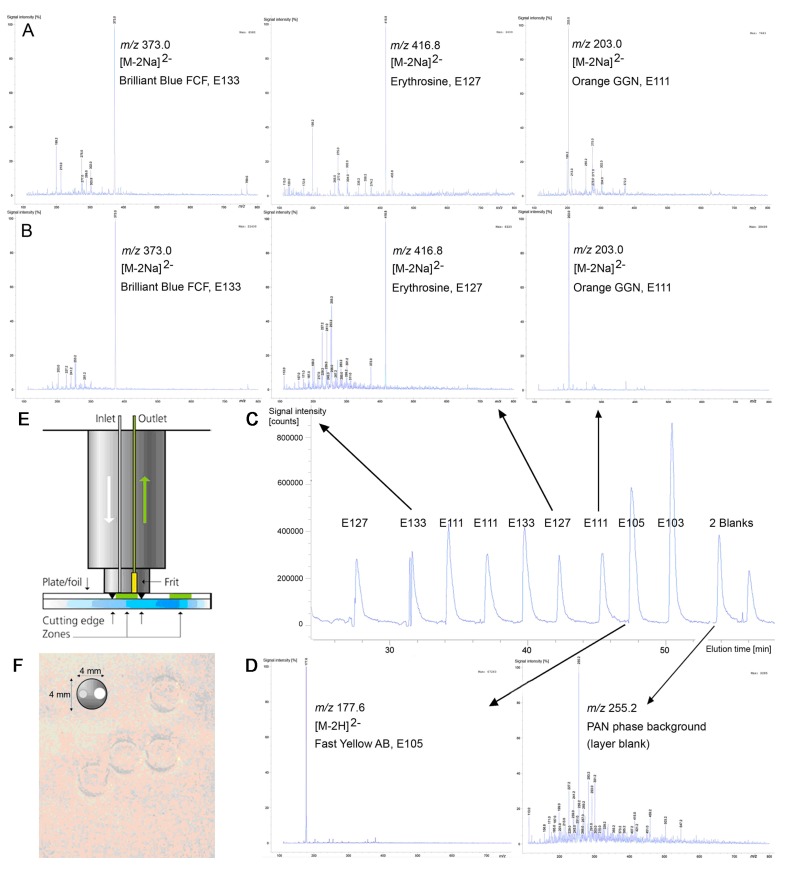
Comparison of HPTLC-ESI-MS spectra for E 133, E 127, and E 111 obtained from HPTLC foils silica gel 60 (**A**) with UTLC-Vis-ESI-MS spectra recorded from electrospun PAN nanofiber phases (**B**); chronogram of elution profiles (**C**) and mass spectra of E 105 and PAN phase background (**D**); scheme of the elution head (**E**) and some fiber mat imprints after elution on the electrospun PAN nanofiber phase (**F**).

For the first time, UTLC-ESI-MS spectra were recorded for the water-soluble food dyes on the electrospun PAN nanofiber phases and compared to those mass spectra obtained from HPTLC foils silica gel 60 (Figure 4A versus Figure 4B) using an elution head-based interface (Figure 4E). Eluents containing methanol and its mixture with water were tested for elution. Using methanol, the highest signal-to-noise ratio was obtained and a water addition for elution of the water-soluble dyes was not indicated. After lifting the elution head, the still wet layer material was not clogging within the cutting edge and removed from the carrier like known for particulate TLC/HPTLC adsorbents. The cutting edge of the elution head only caused an imprint on the flexible aluminum foil carrier after elution of the dyes from the electrospun PAN nanofiber phase (Figure 4F). The elastic fiber mat was still intact and not destroyed by the pressure of the elution head onto the fibers. The elution head distance between adjacent bands was reducible to zero, as there was no damage of the layer and thus no leakage for elution of adjacent zones (Figure 4F). The elution head cleaning between zone elutions was omitted because, firstly, a crossover of dye traces was not evident in the mass spectra for the small given amounts of ca. 20 ng/band each eluted into the single quadrupole MS, and secondly, no clogging of the cutting edge of the elution head had occurred. In contrast to particulate layers that require a drying of wetted adsorbent particles that stick to the cutting edge of the elution head, this was a great advantage. The 4 mm circular elution head was used for elution of the dyes directly into the MS. However, a circular elution head with a diameter of 2 mm (not available) instead of 4 mm would have been suited better for the tiny bands. The many advantages of recording mass spectra from electrospun layers reported in a recent study [11] were confirmed, i.e., the intact layer material after elution, the omission of the elution head cleaning, and the good detectability.

The total ion current (TIC) elution profiles of the eluted zones were clearly visible in the chronogram (Figure 4C). Clear base peaks were obtained in the negative ion mode after elution of the small analyte amounts (about 20 ng/band for all dyes; 9 nL of the 2.5 µg/µL solution) from the electrospun PAN nanofiber phases into the MS, exemplarily shown for E 105, E 111, E 127, and E 133 (Figure 4B,D). In the positive ion mode, the mass signals were marginal. For the dyes investigated, the desodiated molecule of E 103 at *m/z* 293.0 [M−Na]^−^, the twofold deprotonated molecule of E 105 at *m/z* 177.6 [M−2H]^2−^ and for the other dyes (E 111, E 127 and E 133) the twofold desodiated molecules [M−2Na]^2−^ were the dominant and characteristic base peaks (Table 2), as already reported in [17] using HPTLC plates silica gel 60. Other minor signals in the mass spectra of the electrospun layers originated from the plate background. For example, in the mass spectrum of E 127, the electrospun PAN nanofiber background was clearly visible. Of course, at low amounts and/or low elution/ionization properties of an analyte, the phase background is more pronounced in the mass spectrum. The mass spectra obtained from HPTLC foils silica gel 60 showed the same base peaks (Figure 4A versus Figure 4B). Of course, the capability of the detection of dyes is dependent on the mass analyzer used (Orbitrap MS or TOF MS or MS/MS versus single quadrupole MS as used in this study) as well as the mode in which the mass spectra are recorded (selected ion monitoring (SIM) mode versus full scan mode as used in this study). Hence, the capability of detection via UTLC-ESI-MS is expected to be much better when using more efficient mass analyzers or when recording in the SIM mode.

## 4. Conclusions

The electrospun PAN nanofibers were used as the stationary phase for UTLC. As an example, the low nanoliter application via a modified office printer and separation of 5 water-soluble food dyes on the electrospun PAN nanofibrous phase was successfully demonstrated. Figures of merit (plate numbers, resolutions, reproducibilites, and calibration functions) outlined its good performance and suitability for chromatography. Furthermore, mass spectra were successfully recorded from the electrospun PAN nanofiber phases after development, whereby the electrospun layer was not damaged. Only the imprint of the cutting edge of the elution head was visible on the aluminum foil carrier. Further, the elution head cleaning process after each elution was skipped. In contrast, particulate layers require a drying of wetted adsorbent particles that stick to the cutting edge of the elution head and thus allow these particles to be removed. The elution head distance between adjacent bands was reduced to zero as there was no damage of the layer and thus no leakage. Nevertheless, an elution head with a diameter of 2 mm is recommended for the tiny bands. The obtained results suggested that the electrospun PAN nanofibrous phase possesses potential for use as stationary phase for UTLC.

## Figures and Tables

**Table 1:** Performance data of electrospun PAN nanofiber phases: Mean *hR_F_* values of five water-soluble food dyes and their precisions for five different separations as well as the mean plate numbers *N*, linear 4-point calibrations and mean resolutions *R*_S_, calculated between adjacent water-soluble food dyes.

Food Dye	*Mean hR_F_* (*n* = 5)	Precision (%*RSD*, *n* = 5)	Mean N (*n* = 5)	Linear Calibration		Adjacent Food Dyes	*Mean R*_S_	*SD* (*n* = 5)
				*sdv* (Peak Area)	*sdv* (Peak Height)			
**E 102**	25	1	8345	6.0	9.3	**E 102/111**	1.7	0.2
**E 111**	33	4	8286	*	*	**E 111/133**	6.5	0.2
**E 133**	57	14	22,885	3.1	2.4	**E 133/127**	4.6	0.6
**E 127**	73	16	9596	4.5	5.7	**E 127/104**	5.2	0.2
**E 104**	90	1	9497	9.1	4.5	**-**	**-**	**-**
**Mean**	**(25–90)**	**7**	**11,722**	**5.7**	**5.5**	**Mean**	**4.5**	**0.3**

* Lowest two standard levels of the yellow E 111 were < LOQ.

**Table 2:** Structure formulae of the food dyes, their molecular weights, and mass signals on HPTLC foils silica gel 60 versus electrospun PAN nanofiber phases.

Structure Formula *	Food Dye (E Number)	Molecular Weight (g/mol)	Mass Signal *m*/*z* on HPTLC Foils Silica Gel 60	Mass Signal *m*/*z* on Electrospun PAN Nanofibers
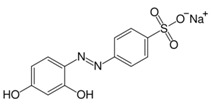	Chrysoine resorcinol (E 103)	316.3	n.d.	293.0 [M−Na]^−^
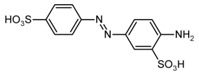	Fast Yellow AB (E 105)	357.4	n.d.	177.6 [M−2H]^2−^
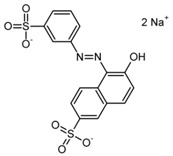	Orange GGN (E 111)	452.4	203.0 [M−2Na]^2−^	203.0 [M−2Na]^2−^
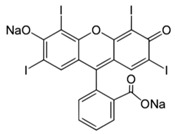	Erythrosine (E 127)	879.9	416.8 [M−2Na]^2−^	416.8 [M−2Na]^2−^
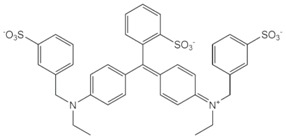	Brilliant Blue FCF (E 133)	792.9	373.0 [M−2Na]^2−^	373.0 [M−2Na]^2−^

n.d. not determined, * www.wikipedia.org.
